# Association Between Incorrect Posture and Adolescent Idiopathic Scoliosis Among Chinese Adolescents: Findings From a Large-Scale Population-Based Study

**DOI:** 10.3389/fped.2020.00548

**Published:** 2020-09-15

**Authors:** Bin Yan, Xinhai Lu, Qihua Qiu, Guohui Nie, Yeen Huang

**Affiliations:** ^1^The First Affiliated Hospital of Shenzhen University, Shenzhen, China; ^2^Department of Spine Surgery, The Shenzhen Second People's Hospital, Shenzhen, China; ^3^Shenzhen Youth Spine Health Center, Shenzhen, China

**Keywords:** adolescent, scoliosis, incorrect posture, risk factor, screening

## Abstract

**Objectives:** Adolescent idiopathic scoliosis (AIS) affects between 1 and 4% of adolescents, and severe curvature may be related to their adverse long-term outcomes. However, whether the change in body appearance is related to AIS remains largely unclear. We aimed to explore the association between incorrect posture and AIS among Chinese adolescents.

**Methods:** Data were collected from a population-based (595,057) school scoliosis screening program in China. A sample of 3,871 adolescents was classified as cases with a diagnosed radiological lateral Cobb angle ≥10°, and 3,987 control subjects with a Cobb angle <10° were randomly selected from the screening system. Adolescents were accessed with demographic information and incorrect posture measured by visual inspection of physical signs, Adam's forward bending test (FBT), and the angle of trunk rotation (ATR). Logistic regression (LR) models were used to examine the associations.

**Results:** Multivariate LR showed that shoulder-height difference, scapula tilt, lumbar concave, and pelvic tilt were associated with AIS. Adolescents with angle of thoracic rotation ≥5° [adjusted odds ratio (AOR) = 5.33–14.67, *P* < 0.001], thoracolumbar rotation ≥5° (AOR = 4.61–5.79, *P* < 0.001), or lumbar rotation ≥5° (AOR = 7.49–7.85, *P* < 0.001) were at especially higher risk for AIS than those with ATR <5°.

**Conclusions:** Incorrect posture may be the potential risk factor for developing AIS, and ATR ≥5° was an important indicator for predicting the occurrence of scoliosis. Early monitoring of incorrect posture for school adolescents should be considered as a routine intervention to effectively identify the progress of scoliosis.

## Introduction

Adolescents idiopathic scoliosis (AIS) is the most common form of spine deformity that has a radiological lateral Cobb angle of at least 10° (1). Evidence showed that AIS affects 1–4% of adolescents in the early stages of puberty, and severe spinal curvature might be associated with their adverse long-term health outcomes (e.g., pulmonary disorders, back pain, disability, psychological effects, and reduced quality of life) (2, 3). In China, there are more than 300 million adolescents aged 10–18 years, and the overall prevalence of AIS was reported to be 5.14% (4), so there could be millions with AIS. Although effective treatment (brace or surgery) has been recommended for AIS in preventing further progression (5, 6), it is believed that early detection of irregular physical signs related to scoliosis enables effective intervention on relatively small curves.

So far, the etiology of AIS is still largely unclear. Some potential influencing factors (such as genetic basis, neurophysiological dysfunction, or skeletal growth) have been identified but not confirmed (2). In previous studies, many researchers have discussed how a patient's body asymmetry [e.g., shoulder imbalance (7), scapular tilt (8), and asymmetric spinal loading (9)] occurred after being diagnosed with scoliosis. However, in our previous large-scale scoliosis screening study (10), we found that the majority of adolescents with scoliosis could be screened for body asymmetry (e.g., shoulder-height difference, scapula prominence, lumbar concave, etc.) before being diagnosed. Therefore, we speculated that some asymmetric manifestations of body structure and function may be recognized before scoliosis occurred.

Incorrect posture refers to an abnormal body state in which the individual's body cannot maintain a standing stability and normal function of tissues and organs in an upright body posture (10, 11). Our previous population-based (595,057) study showed that 65.3% of primary and middle school students were screened to have incorrect posture (10), and incorrect standing posture in adolescents was shown to be associated with spinal pain which may be one of the signs of progressing to scoliosis (12, 13). However, whether incorrect body posture is directly related to scoliosis remains largely unclear.

Therefore, we collected data from the 2019 Chinese School-based Scoliosis Screening Program (CSSSP) to explore the potential association between incorrect posture and AIS. Our findings will help to comprehensively understand the potential risk factors associated with scoliosis and provide targeted intervention strategies for AIS.

## Methods

### Participants and Data Collection

Data of our large-scale population-based (595,057) study was collected from the 2019 CSSSP in southern China, which was an ongoing school-based scoliosis screening program targeted for Chinese children and adolescents (age, 6–18 years old) (14). CSSSP is part of the national public health project, collecting a large population-based scoliosis-related data in southern China every year since 2013, and conducted and administered by the Shenzhen Youth Spine Health Center (SYSHC) of the Shenzhen Second People's Hospital. Detailed sampling method could be found in our previous study (10). Data were collected from February 2019 to January 2020 based on the scoliosis screening network system. Finally, a sample of 3,871 subjects were classified as the AIS group with a diagnosed radiological lateral Cobb angle ≥10°, and 3,987 randomly selected control subjects were categorized as the non-AIS group with a Cobb angle <10°.

### School Scoliosis Screening Program

The screening program for AIS was conducted by the SYSHC of the Shenzhen Second People's Hospital using a national scoliosis screening standardized protocol (GB/T 16133-2014) (15). School screening was performed by two independent experienced rehabilitation therapists using the visual inspection of physical signs, Adam's forward bending test (FBT), and measurement of the angle of trunk rotation (ATR). If the judgments of the two therapists were inconsistent, the third therapist would make the final decision to minimize the subjective bias. More detailed methods and processes for scoliosis screening could be found in our previous published study (10).

### Measures

#### Incorrect Posture

Incorrect posture was assessed by visual inspection, the Adam's FBT, and the ATR. The standard visual inspection was performed in the upright position, and the examiners checked for spine alignment, shoulder asymmetry (e.g., shoulder-height difference), scapula prominence (e.g., scapula tilt), hip and pelvic obliquity (e.g., pelvic tilt), back symmetry (e.g., flat back, thoracic kyphosis), lumbar curvature (e.g., lumbar concave, lumbar kyphosis), distance of hands from the flanks, and length of the lower limbs (16). The Adam's FBT was performed with the student's feet placed together, knees straight, while bending at the hips to nearly 90° with their arms freely hanging forward, palms together (17). Students with any significant physical signs were recorded. The ATR was measured with a scoliometer for quantitative assessment of the angle of thoracic rotation, angle of lumbar rotation, and angle of thoracolumbar rotation (18). Students with an ATR ≥5° or with one or more significant physical signs were identified as having an incorrect posture. When students had an ATR ≥5° or with two or more significant physical signs of scoliosis, they would be rescreened by specially trained physicians and referred for a standing posteroanterior radiograph of the whole spine for final diagnoses (4), and those who were found to have a Cobb angle ≥10° measured by two independent experienced observers would be confirmed as AIS (2).

#### Demographic Information

Demographic characteristics included gender, age, ethnic group, and clinician's recommendations. Students' gender (boys or girls), age (years, calculated from the date of screening minus the date of birth), and ethnic group (“Han” or “minorities”) (19) was obtained based on their identification (ID) card information. Clinician's recommendations were categorized as observation, exercise intervention, brace, and brace or surgery according to the International Society on Scoliosis Orthopedic and Rehabilitation Treatment (SOSORT) guidelines. All the above student information has been linked to the scoliosis screening network system.

### Statistical Analysis

Descriptive analyses were conducted to describe the demographic characteristics and prevalence of incorrect posture among adolescents stratified by AIS, chi-square (χ^2^) test or *t*-test was used to compare the differences between non-AIS and AIS groups. Furthermore, univariate logistic regression (LR) models were applied to preliminarily explore the association between incorrect posture and AIS, and multivariate LR were conducted to test the independent effects of each indicator of incorrect posture. Odds ratios (ORs), adjusted odds ratios (AORs), and 95% confidence intervals (CIs) were obtained from the LR models. Missing data accounted for <1.2% for all relevant variables and were eliminated from the statistical analysis. A two-tailed *P* < 0.05 was considered statistically significant. All statistical analyses were conducted using IBM SPSS version 24.0 (IBM Corp, Armonk, NY, USA).

## Results

### Demographic Characteristics of Participants Stratified by the Occurrence of Adolescent Idiopathic Scoliosis

As shown in Table 1, of the total sample analyzed, 3,871 (49.3%) adolescents were diagnosed with AIS (Cobb ≥10°), 3,987 (50.7%) adolescents were non-AIS (Cobb <10°). AIS was more common in girls than in boys (64.8 vs. 36.1%, *P* < 0.001), and the girls-to-boys ratio was 1.8:1 in AIS group. The mean (standard deviation) age of the AIS group was higher than that in the non-AIS group (13.1 ± 1.9 vs. 12.7 ± 2.0, *P* < 0.001), the proportion of people aged 16–19 years in the AIS group was greater than that in the non-AIS group (13.3 vs. 8.4%, *P* < 0.001). For patients with AIS, 3,127 (80.8%) patients had Cobb angles between 10° and 25°, 742 (19.2%) patients were measured with Cobb angles between 26° and 45°, and patients in these two groups were required to perform exercise intervention and wear braces, respectively. Only two patients with a Cobb angle >45° underwent surgery.

**Table 1:** Demographic characteristics of participants stratified by the occurrence of AIS (*N* = 7,858).

**Variables**	**Non-AIS group** **(Cobb <10°)**	**AIS group** **(Cobb ≥10°)**	***χ^2^*/*t***	***P***
	***n* (%)**	***n* (%)**		
**Total**	3,987 (100.0)	3,871 (100.0)	–	–
**Gender**			647.00	<0.001
Boys	2,548 (63.9)	1,363 (35.2)		
Girls	1,439 (36.1)	2,508 (64.8)		
**Age (years)**			95.64	<0.001
7–12	1,970 (49.4)	1,537 (39.7)		
13–15	1,683 (42.2)	1,819 (47.0)		
16–19	334 (8.4)	515 (13.3)		
**Ethnic group**			0.05	0.819
Han	3,871 (97.1)	3,755 (97.0)		
Minorities	116 (2.9)	116 (3.0)		
**Distribution of Cobb angle**			–	–
<10°	3,987 (100.0)			
10°-25°	–	3,127 (80.8)		
26°-45°	–	742 (19.2)		
>45°	–	2 (<0.001)		
**Clinician's recommendations**			–	–
Observation	3,987 (100.0)	–		
Exercise intervention	–	3,127 (80.8)		
Brace	–	742 (19.2)		
Brace or surgery	–	2 (<0.001)		

*AIS, adolescent idiopathic scoliosis; n, number; SD, standard deviation*.

### Prevalence of Incorrect Posture Among Chinese Adolescents Stratified by the Occurrence of Adolescent Idiopathic Scoliosis

As shown in Table 2, except for lumbar kyphosis, the prevalence of all other indicators of incorrect posture were different between the AIS group and the non-AIS group (*P* < 0.001). Compared to the non-AIS group, the frequencies of adolescents with angles of thoracic rotation ≥5°, thoracolumbar rotation ≥5°, and lumbar rotation ≥5° were significantly greater in the AIS group (*P* < 0.001).

**Table 2:** Prevalence of incorrect posture among Chinese adolescents stratified by the occurrence of AIS (*N* = 7,858).

**Variables**	**Non-AIS group** **(Cobb <10°)**	**AIS group** **(Cobb ≥10°)**	***χ^2^***	***P***
	***n* (%)**	***n* (%)**		
**Total**	3,987 (100.0)	3,871 (100.0)		
Incorrect posture^a^				
**Shoulder-height difference**			1,475.79	<0.001
Normal	3,469 (87.0)	1,796 (46.4)		
Left shoulder height	327 (8.2)	1103 (28.5)		
Right shoulder height	191 (4.8)	972 (25.1)		
**Scapula tilt**			2,460.31	<0.001
Normal	3,405 (85.4)	1,173 (30.3)		
Tilt to the left	379 (9.5)	1,529 (39.5)		
Tilt to the right	203 (5.1)	1,169 (30.2)		
**Lumbar concave**			1,533.17	<0.001
Normal	3,624 (90.9)	1,970 (50.9)		
Left concave	140 (3.5)	794 (20.5)		
Right concave	223 (5.6)	1,107 (28.6)		
**Pelvic tilt**			582.34	<0.001
Normal	3,832 (96.1)	3,039 (78.5)		
Tilt to the left	104 (2.6)	298 (7.7)		
Tilt to the right	51 (1.3)	534 (13.8)		
**Flat back**			21.51	<0.001
Normal	3,979 (99.8)	3,832 (99.0)		
Abnormal	8 (0.2)	39 (1.0)		
**Thoracic kyphosis**			62.52	<0.001
Normal	3,947 (99.0)	3,728 (96.3)		
Abnormal	40 (1.0)	143 (3.7)		
**Lumbar kyphosis**			2.44	0.118
Normal	3,971 (99.6)	3,863 (99.8)		
Abnormal	16 (0.4)	8 (0.2)		
**Angle of thoracic rotation**			1,209.99	<0.001
Normal (ATR: 0°-4°)	3,800 (95.3)	2,474 (63.9)		
Rotate to the left (ATR ≥5°)	80 (2.0)	395 (10.2)		
Rotate to the right (ATR ≥5°)	107 (2.7)	1,002 (25.9)		
**Angle of thoracolumbar rotation**			185.08	<0.001
Normal (ATR: 0°-4°)	3,883 (97.4)	3,484 (90.0)		
Rotate to the left (ATR ≥5°)	40 (1.0)	186 (4.8)		
Rotate to the right (ATR ≥5°)	64 (1.6)	201 (5.2)		
**Angle of lumbar rotation**			1,161.88	<0.001
Normal (ATR: 0°-4°)	3,636 (91.2)	2,237 (57.8)		
Rotate to the left (ATR ≥5°)	271 (6.8)	1,212 (31.3)		
Rotate to the right (ATR ≥5°)	80 (2.0)	422 (10.9)		

*AIS, adolescent idiopathic scoliosis; n, number; ATR, angle of trunk rotation*.

a *Incorrect posture was defined as a participant who was screened out one or more to the following physical signs: shoulder-height difference, scapula tilt, lumbar concave, pelvic tilt, flat back, thoracic kyphosis, lumbar kyphosis, angle of thoracic rotation ≥5°, angle of thoracolumbar rotation ≥5°, angle of lumbar rotation ≥5°*.

### Association Between Incorrect Posture and Adolescent Idiopathic Scoliosis Among Chinese Adolescents

As shown in Table 3, in the univariate LR model (Model 1), gender, age, shoulder-height difference, scapula tilt, lumbar concave, pelvic tilt, thoracic kyphosis, angle of thoracic rotation, angle of thoracolumbar rotation, and angle of lumbar rotation were significantly associated with AIS (*P* < 0.001). In the multivariate LR method (Model 2), except for thoracic kyphosis, the associations of the above indicators with AIS were weakened, but there was still statistical difference (*P* < 0.01). Adolescents with angles of thoracic rotation ≥5°, thoracolumbar rotation ≥5°, and lumbar rotation ≥5° were significantly associated with a higher risk for developing AIS than those with ATR <5° (*P* < 0.001).

**Table 3:** Association between incorrect posture and AIS among Chinese adolescents (*N* = 7,858).

**Variables**	**AIS: Model 1^a^**			**AIS: Model 2^b^**		
	**OR**	**95% CI**	***P***	**AOR**	**95% CI**	***P***
**Gender**						
Boys	1.00			1.00		
Girls	3.26	2.69–3.96	<0.001	1.99	1.42–2.78	<0.001
**Age (years)**	1.14	1.08–1.19	<0.001	1.09	1.02–1.17	<0.001
7–12	1.00			1.00		
13–15	1.38	1.09–1.75	0.007	1.18	0.83–1.69	0.354
16–19	1.99	1.35–2.93	<0.001	1.96	1.12–3.42	0.018
**Ethnic group**						
Han	1.00			1.00		
Minorities	0.98	0.82–1.13	0.889	0.99	0.86–1.12	0.631
**Incorrect posture**						
**Shoulder-height difference**						
Normal	1.00			1.00		
Left shoulder height	6.56	4.92–8.74	<0.001	2.63	1.53–4.53	<0.001
Right shoulder height	9.81	7.93–11.89	<0.001	3.72	1.99–6.97	<0.001
**Scapula tilt**						
Normal	1.00			1.00		
Tilt to the left	11.71	9.89–12.41	<0.001	2.34	1.38–3.97	<0.001
Tilt to the right	16.55	13.75–18.30	<0.001	2.89	1.57–5.30	0.001
**Lumbar concave**						
Normal	1.00			1.00		
Left concave	10.56	8.09–12.72	<0.001	3.62	1.88–6.96	<0.001
Right concave	9.15	7.62–11.66	<0.001	3.81	2.30–6.28	<0.001
**Pelvic tilt**						
Normal	1.00			1.00		
Tilt to the left	3.66	2.26–5.94	<0.001	0.35	0.17–0.74	0.006
Tilt to the right	12.60	8.91–14.99	<0.001	1.42	0.60–3.35	0.420
**Flat back**						
Normal	1.00			1.00		
Abnormal	2.59	0.92–3.32	0.852	1.28	0.76–3.19	0.445
**Thoracic kyphosis**						
Normal	1.00			1.00		
Abnormal	3.82	2.82–5.03	<0.001	1.98	0.83–4.37	0.117
**Lumbar kyphosis**						
Normal	1.00			1.00		
Abnormal	0.51	0.09–2.77	0.431	0.10	0.01–1.77	0.990
**Angle of thoracic rotation**						
Normal (ATR: 0°-4°)	1.00			1.00		
Rotate to the left (ATR ≥5°)	7.55	5.50–8.66	<0.001	5.33	2.51–11.32	<0.001
Rotate to the right (ATR ≥5°)	14.41	12.34–16.23	<0.001	14.67	7.69–17.97	<0.001
**Angle of thoracolumbar rotation**						
Normal (ATR: 0°-4°)	1.00			1.00		
Rotate to the left (ATR ≥5°)	8.11	6.47–9.57	<0.001	5.79	2.30–14.58	<0.001
Rotate to the right (ATR ≥5°)	5.60	3.96–7.60	<0.001	4.61	2.06–10.31	<0.001
**Angle of lumbar Rotation**						
Normal (ATR: 0°-4°)	1.00			1.00		
Rotate to the left (ATR ≥5°)	7.25	5.38–9.77	<0.001	7.49	4.79–11.71	<0.001
Rotate to the right (ATR ≥5°)	8.52	6.09–10.26	<0.001	7.85	3.68–16.73	<0.001

*AIS, adolescent idiopathic scoliosis; OR, odds ratio; AOR, adjusted odds ratio; CI, confidence interval; ATR, angle of trunk rotation*.

a *Univariate logistic regression model*.

b *Multivariate logistic regression model*.

## Discussion

In our 2019 CSSSP, adolescents participating in the school screening would undergo a rigorous physical examination by two independent experienced therapists, and scoliosis patients could be effectively identified by some signs of incorrect posture assessed by the visual inspection of physical signs, the Adam's FBT, and measurement of the ATR. Using a large-scale population-based (595,057) dataset, we further clarified the role of incorrect posture in identifying AIS and non-AIS groups and found that ATR ≥5° might have the strongest associations with the occurrence of scoliosis in adolescents. This indicated that incorrect posture could be a critical indicator for school scoliosis screening, especially the indicator of ATR. Our findings could help resolve the problem of low positive predictive value (PPV) and high referral rate in school-based screening for scoliosis.

Using incorrect posture as the indicators for screening scoliosis, we found that the prevalence of shoulder-height difference, scapula tilt, lumbar concave, pelvic tilt, flat back, and thoracic kyphosis was significantly higher in adolescents diagnosed with AIS than those with non-AIS, which were consistent with the previous research (20). Furthermore, multivariate LR model showed that shoulder-height difference, scapula tilt, and lumbar concave were independently associated with AIS. Using biomechanics and three-dimensional spatial positioning methods, some researchers speculated the alterations of shoulder, scapula, and lumbar spine could be considered as adaptive compensation or muscle activation strategies in AIS patients (21–23). Interestingly, we found that a pelvic tilt to the left (AOR = 0.35, 95% CI: 0.17–0.74) seems to be a protective factor for scoliosis. This may be related to the fact that most scoliosis occurs on the right side; in order to maintain the balance of the body in a sagittal standing posture, the pelvis of the individual is prone to tilt to the left under the effect of adaptive compensation (24).

The validity and reliability of using the scoliometer to measure ATR for scoliosis screening have been verified (25, 26), but few studies have explored the magnitude of the association between ATR and scoliosis. We filled the gaps in this field and found that the angles of thoracic rotation ≥5°, thoracolumbar rotation ≥5°, and lumbar rotation ≥5° were significantly associated with a higher risk for AIS than those with ATR <5°, in which adolescents whose angle of thoracic rotation to the right ≥5° were 10 times more likely to develop scoliosis than adolescents with ATR <5°. Studies from Pratt et al. (27) and Scutt et al. (28) both showed that a larger ATR was related to more severe scoliosis. A recent cross-sectional study indicated that rotation of the thoracic and thoracolumbar spine appeared to be the most differentiating in the diagnosis of AIS, and mild scoliosis could be found even in adolescents with a lower degree of trunk rotation (4°-6°) (29). Our research supported the results of previous studies and further quantified the relationship between ATR and AIS, which could provide an objective reference for more accurate identification of scoliosis patients in large-scale school screening.

The benefits and harms of school screening for AIS remain controversial. This is mainly related to the relatively low PPV for identifying patients and that leads to over-referral of adolescents who do not require follow-up or radiography (3). In order to solve this problem, we put forward a new concept of “incorrect posture,” which includes 10 indicators related to changes in body appearance that can be measured by visual inspection of physical signs, the Adam's FBT, and the ATR. Using this screening method, our previous findings showed a PPV of 83.8% for identifying AIS patients with a Cobb angle ≥10° in a large-scale (961,169 adolescents) school scoliosis screening (14), which was higher than the results of the largest cohort study conducted in Hong Kong (16). This showed the feasibility and effectiveness of using incorrect posture as the identification indicator for school scoliosis screening in China.

The present study had several limitations that were worth noting. First, due to the cross-sectional design of the study, it was difficult to make causal inferences, so the possibility of reverse association might exist. Second, although each indicator of incorrect posture was judged by two independent therapists, it was still difficult to completely avoid subjective measurement bias. Third, due to the large number of adolescents needed to be screened, other influencing factors (e.g., genetics, hormone, or nutritional status) related to AIS had not been fully investigated, which might overestimate the magnitude of the association between incorrect posture and AIS. Fourth, since the nature of the cross-sectional data, we could only obtain limited information about the condition of AIS patients, the longitudinal relationship between incorrect posture and the development of AIS could not be discussed. Future prospective cohort studies would help fill the gaps in this research area. Fifth, our data mainly came from subjective physical examinations; although the measurement results in the study were assessed by two independent observers, potential measurement bias between observers for the severity of incorrect posture might exist.

## Conclusion

Using a population-based screening dataset, we found that incorrect posture was associated with AIS among Chinese adolescents, and ATR ≥5° could be considered as the best indicator to identify the occurrence of scoliosis. Early monitoring and identification of bad postures could be considered as a feasible and effective targeted intervention for preventing future AIS. More longitudinal studies to clarify the causal relationship between incorrect posture and scoliosis are needed.

## Data Availability Statement

The datasets supporting the conclusions of this article will be made available by the authors upon reasonable request.

## Ethics Statement

This study was conducted in accordance with the Declaration of Helsinki and was approved by the Shenzhen Municipal Health Commission Institutional Review Board. Written informed consent was obtained from the individual(s), and minor(s)' legal guardian/next of kin, for the publication of any potentially identifiable images or data included in this article.

## Author Contributions

YH and GN designed and supervised the study. BY and XL collected the screening data. XL and QQ carried out the statistical analysis. BY wrote the original draft. YH and GN reviewed and corrected the revised manuscript. All authors contributed to the article and approved the submitted version.

## Conflict of Interest

The authors declare that the research was conducted in the absence of any commercial or financial relationships that could be construed as a potential conflict of interest.
